# Lower Urinary Tract Infection and Subsequent Risk of Prostate Cancer: A Nationwide Population-Based Cohort Study

**DOI:** 10.1371/journal.pone.0168254

**Published:** 2017-01-03

**Authors:** Chao-Yueh Fan, Wen-Yen Huang, Kuen-Tze Lin, Chun-Shu Lin, Hsing-Lung Chao, Jen-Fu Yang, Cheng-Li Lin, Chia-Hung Kao

**Affiliations:** 1 Department of Radiation Oncology, Tri-Service General Hospital, National Defense Medical Center, Taipei, Taiwan; 2 Institute of Clinical Medicine, National Yang-Ming University, Taipei, Taiwan; 3 Management Office for Health Data, China Medical University Hospital, Taichung, Taiwan; 4 College of Medicine, China Medical University, Taichung, Taiwan; 5 Graduate Institute of Clinical Medical Science and School of Medicine, College of Medicine, China Medical University, Taichung, Taiwan; 6 Department of Nuclear Medicine and PET Center, China Medical University Hospital, Taichung, Taiwan; 7 Department of Bioinformatics and Medical Engineering, Asia University, Taichung, Taiwan; Universite Clermont Auvergne, FRANCE

## Abstract

**Purpose:**

We investigated whether lower urinary tract infection (LUTI), including cystitis or urethritis, is associated with an increased risk of developing prostate cancer (PCa), in a nationwide population-based cohort study.

**Methods:**

We identified 14,273 men newly diagnosed with LUTI (9347 with cystitis, and 4926 with urethritis) between 1998 and 2011, from the Taiwan Longitudinal Health Insurance Database 2000. Each patient was randomly frequency-matched with 4 men without LUTI, based on age and index year of diagnosis. Cox’s proportional hazard regression analysis was performed to estimate the effect of LUTI on the PCa risk.

**Results:**

The risk of developing PCa was significantly higher in the cystitis cohort (adjusted HR = 1.46, 95% CI = 1.20–1.78) and in the urethritis cohort (adjusted HR = 1.72, 95% CI = 1.26–2.34) than in the group without LUTI. Further analyses indicated that patients with more than 5 medical visits for LUTI per year had a significantly greater risk of developing PCa.

**Conclusion:**

We found that cystitis or urethritis may play an etiological role in the development of PCa in Taiwanese men, particularly in those with repeated medical visits for cystitis or urethritis. Further studies are warranted on the association between LUTI and PCa in other countries, particularly where the prevalence of PCa is high.

## Introduction

Prostate cancer (PCa) is the second most common cancer worldwide in men, with an incidence of 31.1 per 100,000, and it is the fifth most common cause of cancer-related death [1]. Except for increasing age, family history and race [2], the etiological factors triggering PCa have yet to be fully identified. There is increasing evidence in the literature suggesting that inflammation may contribute to prostate carcinogenesis [3]. Inflammation of the prostate, resulting from exposure to microbial agents, stimulates the production of inflammatory cytokines and reactive oxygen species, leading to increased cellular proliferation and, possibly, to carcinogenesis [4, 5]. To date, most studies have focused on the role of prostatitis and sexually transmitted diseases (STDs) in the development of PCa [6–8]. Two meta-analyses have concluded that prostatitis or STDs, which may cause prostatic inflammation, increase the risk of developing PCa [7, 8].

Urinary tract infection is a significant health care burden and the overall lifetime prevalence in men in the United States, between 1988 and 1994, was estimated at 13,689/100,000 [9, 10]. Urinary tract infection is defined as the presence of microbial pathogens in the urethra or bladder (lower urinary tract), or in the ureter and pelvis of the kidney (upper urinary tract). The prostate is located in the pelvis, adjacent to the bladder and surrounding segments of the urethra. Theoretically, lower urinary tract infection (LUTI), such as urethritis and cystitis, may cause inflammation of the prostate and play a role in the development of PCa. However, reports of the association between PCa and LUTI remain scarce. Only two studies have examined the association between PCa and, respectively, cystitis and urethritis [11, 12] but the role played by these infections is unknown. These findings remain controversial because both reports were based on case-control studies using in-person interviews, which are potentially susceptible to recall, selection and interview bias. To clarify this issue, we investigated the PCa risk for patients with cystitis or urethritis in Taiwan, using a population-based cohort study.

The prevalence of PCa is approaching epidemic levels in the United States and Western countries [13]. In the Far East, the incidence of PCa is increasing [14, 15]. According to the 2011 cancer report released by the Taiwan Department of Health, the incidence of PCa in men was 39.7 per 100,000 and it is the seventh leading cause of death from cancer in men in Taiwan [16]. Therefore, there is a critical need for a greater understanding of the etiological factors triggering PCa. The Taiwan National Health Insurance (NHI) program was implemented in 1995, with 97% of the hospitals and clinics throughout Taiwan under contract with the system by the end of 1996 [17]. By 1998, the health care of almost 99% of the population of Taiwan was covered by the NHI. The NHI patient records provide a unique opportunity for a nationwide investigation to examine our hypothesis that urethritis or cystitis in men is associated with an increased risk of developing PCa.

## Methods

### Data Source

The Taiwan National Health Research Institute set up and manages the National Health Insurance Research Database (NHIRD), which includes reimbursement claims data of 23.74 million citizens for the Taiwan NHI program. The data in our retrospective cohort study were obtained from the Longitudinal Health Insurance Database 2000 (LHID 2000), a subset of the NHIRD [18]. The LHID 2000 was randomly sampled from the Registry for Beneficiaries and contains original claims data of one million beneficiaries enrolled in 2000. The details of the NHI program and LHID 2000 have been described in previous studies [19, 20]. The diagnoses in the LHID 2000 are coded on the basis of the International Classification of Diseases, Ninth Revision, Clinical Modification (ICD-9-CM).

### Ethics Statement

The NHIRD encrypts patient personal information to protect privacy and provides researchers with anonymous identification numbers associated with relevant claims information, including sex, date of birth, medical services received, and prescriptions. Therefore, patient consent is not required to access the NHIRD. This study was approved to fulfill the condition for exemption by the Institutional Review Board (IRB) of China Medical University (CMUH104-REC2-115). The IRB also specifically waived the consent requirement.

### Data Availability Statement

All data and related metadata were deposited in an appropriate public repository in the National Health Research Institutes (NHRI). The data on the study population that were obtained from the NHIRD (http://nhird.nhri.org.tw/en/index.html) are maintained in the NHIRD (http://nhird.nhri.org.tw/). The use of NHIRD is limited to research purposes only. Applicants must follow the Computer-Processed Personal Data Protection Law (http://www.winklerpartners.com/?p=987) and related regulations of National Health Insurance Administration and NHRI, and an agreement must be signed by the applicant and his/her supervisor upon application submission. All applications are reviewed for approval of data release.

### Sampled Participants

Patients newly diagnosed with LUTI between 1998 and 2011 (age ≥20 years) were divided into two groups, cystitis (ICD-9-CM code 595) and urethritis (ICD-9-CM code 597). The date of the first medical visit of a LUTI patient registered was defined as the index date. A non-LUTI cohort randomly selected from the LHID 2000 was frequency matched with the LUTI cohort at a 4:1 ratio based on age (every 5-year span), and year of LUTI diagnosis. Patients with a history of cancer (ICD-9-CM codes 140–208) before the index date or aged less than 20 years were excluded. Subjects were followed-up until diagnosis of PCa (ICD-9-CM code 185), death, withdrawal from the NHI program, or December 31, 2011.

### Socio-demographic variables, Comorbidity and Examination

The socio-demographic variables examined in this cohort study include age, occupation and urbanization level. A detailed description of occupation and urbanization level has been presented in the previous article [21]. The following diagnoses were recorded in order to establish the baseline comorbidity history for each participant: hyperlipidemia, diabetes, hypertension, benign prostatic hyperplasia (BPH), urinary calculi, obesity, asthma, coronary artery disease (CAD), chronic obstructive pulmonary disease (COPD), stroke, alcohol-related illness, prostatitis and STDs. The STDs included genital herpes, gonococcal infections, syphilis, and chlamydia trachomatis. PCa screening and diagnostic procedures of transurethral resection of the prostate (TURP) and prostate biopsy were also noted. We tabulated the ICD-9-CM codes of the comorbidities, and ICD-9-procedure codes of TURP and prostate biopsy in Table 1.

**Table 1:** Comparison of demographics and comorbidity between patients with lower urinary tract infection and controls.

	Lower urinary tract infection								p-value
	Cystitis (N = 9347)		Urethritis (N = 4926)		Total (N = 14, 273)		Control (N = 57,092)		
	n	%	n	%	n	%	n	%	
**Age, years**									0.99
≤34	1346	14.4	1485	30.2	2831	19.8	11324	19.8	
35–49	2261	24.2	1412	28.7	3673	25.7	14692	25.7	
50–64	2235	23.9	994	20.2	3229	22.6	12916	22.6	
>65	3505	37.5	1035	21.0	4540	31.8	18160	31.8	
Mean (SD) ^#^	56.1	17.4	47.7	17.6	53.2	18.0	52.6	18.1	0.001
**Occupation**									<0.001
White collar	3978	42.6	2482	50.4	6460	45.3	27945	49.0	
Blue collar	3666	39.2	1626	33.0	5292	37.1	19012	33.3	
Others^‡^	1703	18.2	818	16.6	2521	17.7	10135	17.8	
**Urbanization level**^§^									<0.001
1 (highest)	2475	26.5	1396	28.3	3871	27.1	16087	28.2	
2	2538	27.2	1413	28.7	3951	27.7	16197	28.4	
3	1625	17.4	976	19.8	2601	18.2	10675	18.7	
4	2709	29.0	1141	23.2	3850	27.0	14133	24.8	
**Comorbidity (ICD-9-CM codes)**									
Hyperlipidemia (272)	2112	22.6	842	17.1	2954	20.7	8653	15.2	<0.001
Diabetes (250)	973	10.4	360	7.31	4415	7.73	1333	9.34	<0.001
Hypertension (401–405)	3851	41.2	1380	28.0	17025	29.8	5231	36.7	<0.001
BPH (600.0)	1034	11.1	356	7.23	1390	9.74	1770	3.10	<0.001
Urinary calculi (592.0, 592.1, 594.0, 594.1)	1158	12.4	379	7.69	1537	10.8	2116	3.71	<0.001
Obesity (278)	68	0.73	23	0.47	91	0.64	293	0.51	0.07
Asthma (493)	847	9.06	333	6.76	1180	8.27	3153	5.52	<0.001
CAD (410–414)	2138	22.9	737	15.0	2875	20.1	8305	14.6	<0.001
COPD (491, 492, 496)	1748	18.7	651	13.2	2399	16.8	6917	12.1	<0.001
Stroke (430–438)	650	6.95	203	4.12	853	5.98	2495	4.37	<0.001
Alcohol-related illness (291, 303, 305, 571.0, 571.1, 571.2, 571.3, 790.3, A215, and V11.3)	446	4.77	225	4.57	671	4.70	1945	3.41	<0.001
Prostatitis (601)	569	6.09	369	7.49	938	6.57	605	1.06	<0.001
STDs (054.1, 098, 091, 096, 099.5)	46	0.49	73	1.48	119	0.83	66	0.12	<0.001
**Screening/diagnostic procedures (ICD-9-procedure codes)**									
Prostate biopsy (60.11, 60.12)	628	6.72	173	3.51	801	5.61	908	1.59	<0.001
TURP (60.29)	288	3.08	70	1.42	358	2.51	657	1.15	<0.001

Chi-square test compared to total SD;

^#^: T-test.

^‡^ Other occupations included primarily retired, unemployed, or low-income populations.

^§^: Urbanization was categorized into 4 levels, according to the population density of the residential area, with level 1 as the most urbanized area and level 4 as the least urbanized.

Abbreviation: SD, standard deviation; ICD-9-CM, International Classification of Diseases, Ninth Revision, Clinical Modification; BPH, benign prostatic hyperplasia; CAD, coronary artery disease; COPD, chronic obstructive pulmonary disease; STDs, sexually transmitted diseases; TURP, transurethral resection of the prostate.

### Statistical Analysis

The demographic and clinical characteristics of the LUTI (cystitis and urethritis) and non-LUTI cohorts, including occupation, urbanization level, comorbidities, and PCa screening and diagnostic procedures, were compared using the Chi-square test. For continuous variables such as age, the Student’s *t*-test was used to compare the LUTI and non-LUTI cohorts. The cumulative incidence of PCa in the three cohorts was calculated using the Kaplan-Meier method and difference was evaluated by using the log-rank test. We calculated the incidence density rate of follow up (per 1000 person-years) for each cohort. Univariate and multivariate Cox proportional hazard models were applied to measure the hazard ratios (HRs) and 95% confidence intervals (CIs) of PCa for comparisons between the cystitis and urethritis cohort and the non-LUTI cohort. The multivariate models were simultaneously adjusted for age, occupation, urbanization level and the following comorbidities: hyperlipidemia, diabetes, hypertension, BPH, urinary calculi, obesity, asthma, CAD, COPD, stroke, alcohol-related illness, prostatitis, and STDs, as well as for PCa screening and diagnostic procedures of TURP and prostate biopsy. Further analyses were performed to assess the dose response of LUTI on the risk of developing PCa, according to the number of medical visits for LUTI. We used Cox proportional hazards model to estimate PCa incidence in study cohorts by follow-up year and calculated hazard ratio of developing PCa for study cohorts by follow-up year. All analyses were performed using SAS statistical software (version 9.4 for Windows; SAS Institute, Inc., Cary, NC, USA). A P-value < 0.05 was considered significant for a two-tailed test.

## Results

**Fig 1:**
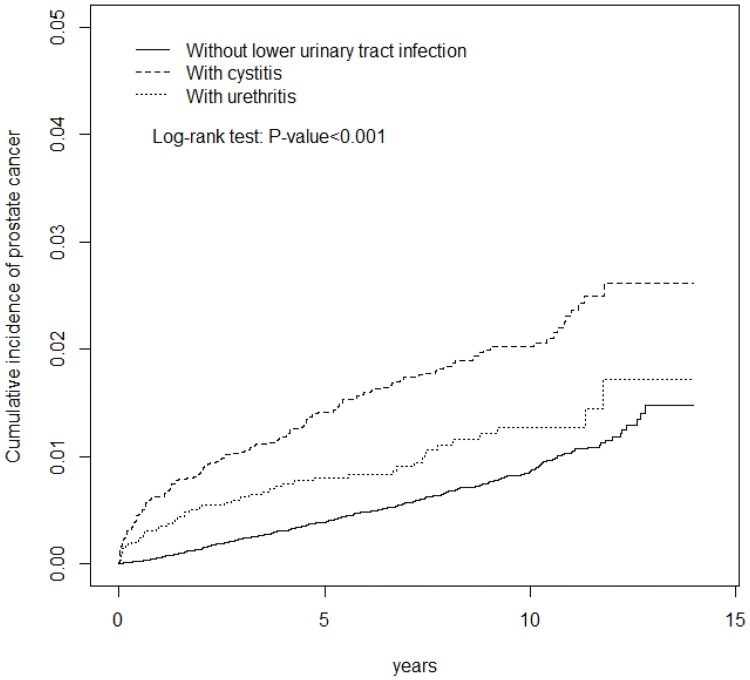
Comparison of the cumulative incidence of prostate cancer (determined by the Kaplan-Meir method) among the cystitis, urethritis and without urinary tract infection cohort groups.

This study included 14,273 patients with newly diagnosed LUTI (9347 cystitis, and 4926 urethritis) between 1998 and 2011 (Table 1). Mean age was 53.2 years for the LUTI cohort and 52.6 years for the non-LUTI cohort, with 68.1% of patients aged <65 years. The urethritis patients were 8.4 years younger than the cystitis patients (56.1 vs. 47.7 years). Subjects were white-collar workers (45.3% LUTI vs 49.0% non-LUTI), living in urbanized areas (54.8% LUTI vs 56.6% non-LUTI). Comorbidities, except for obesity, and PCa screening and diagnostic procedures with prostate biopsy and TURP were more frequent in the LUTI than in the non-LUTI cohort (P < 0.001). There was an increased prevalence of comorbidities, except for prostatitis and STDs, and PCa screening and diagnostic procedures with prostate biopsy and TURP in the cystitis patients compared to the urethritis patients. As shown in Fig 1, the highest cumulative incidence of PCa was observed in the cystitis cohort (log rank test, P < 0.001) by the end of the follow-up period.

The overall incidence density of PCa was significantly higher in the cystitis and urethritis cohort than in the non-LUTI cohort (2.40 vs. 1.42 vs. 0.88 per 1000 person-years, respectively) (Table 2). The risk of PCa was 1.46-fold higher (95% CI = 1.20–1.78) in the cystitis cohort and 1.72-fold higher (95% CI = 1.26–2.34) in the urethritis cohort than in the non-LUTI cohort. The age-specific analysis demonstrated a 1.65-fold higher risk of developing PCa in subjects aged ≤64 years in the cystitis cohort than in the non-LUTI cohort (95% CI = 1.08–2.53). We observed a higher risk of PCa in subjects >65 years in the cystitis cohort (adjusted HR = 1.42, 95% CI = 1.14–1.77) and in the urethritis cohort (adjusted HR = 1.86, 95% CI = 1.30–2.65) than in the non-LUTI cohort.

**Table 2:** Comparison of prostate cancer incidence density and hazard ratio in the study cohorts.

	Control		Cystitis		Crude HR*	Adjusted HR^†^	p-value	Urethritis		Crude HR*	Adjusted HR^†^	p-value
	Case	Rate^#^	Case	Rate^#^	(95% CI)	(95% CI)		Case	Rate^#^	(95% CI)	(95% CI)	
**All**	356	0.88	161	2.40	2.71(2.25, 3.26)	1.46(1.20, 1.78)	<0.001	49	1.42	1.61(1.19, 2.17)	1.72(1.26, 2.34)	<0.001
**Age, years**												
≤64	76	0.26	37	0.81	3.06(2.07, 4.54)	1.65(1.08, 2.53)	0.02	13	0.45	1.77(0.98, 3.19)	1.60(0.87, 2.96)	0.13
>65	280	2.51	124	5.71	2.28(1.84, 2.81)	1.42(1.14, 1.77)	0.002	36	6.08	2.41(1.71, 3.41)	1.86(1.30, 2.65)	<0.001
**Occupation**												
White collar	134	0.67	55	1.90	2.83(2.07, 3.88)	1.55(1.11, 2.16)	0.01	16	0.90	1.36(0.81, 2.28)	1.80(1.04, 3.11)	0.04
Blue collar	133	1.00	74	2.80	2.81(2.12, 3.74)	1.50(1.10, 2.03)	0.01	22	1.98	1.99(1.27, 3.12)	1.78(1.11, 2.85)	0.02
Others^‡^	89	1.29	32	2.71	2.10(1.40, 2.15)	1.17(0.77, 1.79)	0.46	11	1.92	1.50(0.80, 2.80)	1.55(0.81, 2.96)	0.18
**Urbanization level**												
1 (highest)	110	0.96	42	2.38	2.48(1.73, 3.53)	1.36(0.94, 1.98)	0.11	10	1.00	1.04(0.55, 1.99)	1.77(0.91, 3.46)	0.09
2	87	0.76	38	2.06	2.73(1.87, 4.00)	1.51(1.01, 2.26)	0.04	11	1.10	1.47(0.78, 2.74)	1.50(0.79, 2.87)	0.22
3	63	0.84	21	1.76	2.11(1.29, 3.45)	1.21(0.72, 2.04)	0.48	9	1.34	1.61(0.80, 3.23)	1.22(0.55, 2.68)	0.62
4	96	0.98	60	3.13	3.20(2.31, 4.41)	1.69(1.20, 2.38)	0.003	19	2.41	2.46(1.50, 4.02)	2.27(1.37, 3.79)	0.002
**Comorbidity**^**&**^												
No	87	0.36	30	3.20	3.10(2.04, 4.69)	1.81(1.17, 2.79)	0.008	7	0.38	1.06(0.49, 2.29)	2.39(1.10, 5.23)	0.03
Yes	269	1.66	131	0.38	1.93(1.57, 2.38)	1.44(1.16, 1.79)	<0.001	42	2.56	1.57(1.14, 2.18)	1.73(1.25, 2.40)	0.001
Prostatitis												
No	342	0.86	146	2.30	2.69(2.21, 3.26)	1.48(1.21, 1.81)	<0.001	39	1.21	1.42(1.02, 1.98)	1.70(1.21, 2.38)	0.002
Yes	14	4.52	15	4.07	0.92(0.44, 1.91)	1.16(0.54, 2.49)	0.70	10	4.25	0.97(0.43, 2.19)	1.67(0.70, 3.98)	0.25
**Screening/diagnostic procedures**												
**Prostate biopsy**												
No	199	0.50	98	1.50	3.01(2.36, 3.83)	1.74(1.34, 2.25)	<0.001	27	0.79	1.58(1.06, 2.37)	1.53(1.01, 2.31)	0.046
Yes	157	34.5	63	35.0	1.02(0.76, 1.36)	0.99(0.73, 1.34)	0.96	22	54.1	1.57(1.00, 2.45)	1.57(0.98, 2.49)	0.06
**TURP**												
No	285	0.72	117	1.86	2.58(2.08, 3.20)	1.52(1.22, 1.90)	<0.001	35	1.05	1.46(1.03, 2.07)	1.64(1.15, 2.35)	0.007
Yes	71	10.4	44	10.2	0.98(0.67, 1.42)	0.96(0.65, 1.42)	0.039	14	12.3	1.17(0.66, 2.08)	1.18(0.65, 2.14)	0.28

Rate^#^, incidence rate, per 1,000 person-years;

Crude HR*, relative hazard ratio, per 10,000 person-years;

Adjusted HR^†^: multivariate analysis including age, occupation, urbanization level, comorbidity of hyperlipidemia, diabetes, hypertension, benign prostatic hyperplasia, urinary calculi, obesity, asthma, coronary artery disease, chronic obstructive pulmonary disease, stroke, alcohol-related illness, prostatitis, and sexually transmitted diseases, and prostate cancer screening and diagnostic procedures with prostate biopsy and transurethral resection of the prostate;

^‡^ Other occupations included primarily retired, unemployed, or low-income populations.

Comorbidity^&^: The comorbidity group was defined by the presence of at least one of the following conditions: hyperlipidemia, diabetes, hypertension, benign prostatic hyperplasia, urinary stones, obesity, asthma, coronary artery disease, chronic obstructive pulmonary disease, stroke, alcohol-related illness, prostatitis, and sexually transmitted diseases.

The occupation-specific analyses showed that the risk of PCa was higher in the cystitis cohort and in the urethritis cohort than in the non-LUTI cohort for white collar jobs and blue-collar workers. Among subjects living in the second urbanization level, patients with cystitis had a significantly higher risk of PCa than those without LUTI. The risk of PCa for subjects living in the lowest urbanization level was higher in the cystitis and urethritis cohort than in the non-LUTI cohort. Interestingly, LUTI patients without comorbidity demonstrated a higher risk of PCa than the non-LUTI cohort without any comorbidity (aHR in cystitis cohort = 1.81, 95% CI = 1.17–2.79; aHR in urethritis cohort = 2.39, 95% CI = 1.10–5.23). We excluded patients with prostatitis, and the remainders with LUTI still had an increased risk of developing PCa compared with non-LUTI cohort (aHR in cystitis cohort = 1.48, 95% CI = 1.21–1.81; aHR in urethritis cohort = 1.70, 95% CI = 1.21–2.38). In the subgroup without prostate biopsy and TURP, the risk of developing PCa was significantly higher in LUTI patients than in the non-LUTI cohort (cystitis cohort: aHR in prostate biopsy = 1.74, 95% CI = 1.34–2.25, aHR in TURP = 1.52, 95% CI = 1.22–1.90; urethritis cohort: aHR in prostate biopsy = 1.53, 95% CI = 1.01–2.31, aHR in TURP = 1.64, 95% CI = 1.15–2.35).

Patients in the cystitis cohort with >5 medical visits for cystitis had a significantly higher risk of PCa (aHR = 9.25, 95% CI = 6.94–12.3) than non-LUTI cohorts (Table 3). The urethritis patients with >5 medical visits for urethritis had the highest risk of PCa (aHR = 9.68, 95% CI = 5.81–16.1).

**Table 3:** The risk of prostate cancer according to the average frequency of medical visits for lower urinary tract infection using Cox proportional hazard regression.

Average frequency for medical visit, per years	Event	Person-years	IR	Adjusted HR^†^ (95% CI)	p-value
**Cystitis**					
None	356	402785	0.88	1.00(Reference)	
≤3	59	60325	0.98	0.74(0.56, 1.00)	0.03
4–5	28	4464	6.27	1.83(1.23, 2.73)	0.003
>5	74	2394	30.9	9.25(6.94, 12.3)	<0.001
p for trend				<0.001	
**Urethritis**					
None	356	402785	0.88	1.00(Reference)	
≤3	19	31905	0.60	0.94(0.58, 1.50)	0.78
4–5	7	1756	3.99	1.90(0.88, 4.08)	0.10
>5	23	906	25.4	9.68(5.81, 16.1)	<0.001
p for trend				<0.001	

Adjusted HR†: multivariate analysis including age, occupation, urbanization level, comorbidity of hyperlipidemia, diabetes, hypertension, benign prostatic hyperplasia, urinary calculi, obesity, asthma, coronary artery disease, chronic obstructive pulmonary disease, stroke, alcohol-related illness, prostatitis, and sexually transmitted diseases, and prostate cancer screening and diagnostic procedures with prostate biopsy and transurethral resection of the prostate.

We used sensitivity analysis to validate the association between LUTI occurrence and developing PCa risk in the study population with different follow-up durations (Table 4). When the follow-up time was more than 1 year, the LUTI cohort had an increased risk of PCa compared with the comparison cohort (aHR = 1.46, 95% CI = 1.19–1.80). The risk in the LUTI cohort was not significant when follow-up time was more than 4 years (aHR = 1.24, 95% CI = 0.94–1.65).

**Table 4:** Cox proportional hazards model estimated developing prostate cancer incidence in study cohorts by follow-up year and hazard ratio of developing prostate cancer for study cohorts.

Follow-up time	Control		Lower urinary tract infection		Crude HR*	Adjusted HR^†^	p-value
	Case	Rate^#^	Case	Rate^#^	(95% CI)	(95% CI)	
≥ 1 year	323	0.81	135	1.33	1.65(1.35, 2.02)	1.46(1.19, 1.80)	<0.001
≥ 2 year	282	0.71	108	1.08	1.51(1.21, 1.89)	1.34(1.07, 1.69)	0.01
≥ 3 year	234	0.60	88	0.90	1.48(1.16, 1.89)	1.35(1.04, 1.74)	0.02
≥ 4 year	202	0.54	72	0.76	1.40(1.07, 1.84)	1.24(0.94, 1.65)	0.13
≥ 5 year	168	0.47	54	0.60	1.26(0.93, 1.72)	1.12(0.81, 1.54)	0.49

Rate^#^, incidence rate, per 1,000 person-years;

Crude HR*, relative hazard ratio, per 10,000 person-years;

Adjusted HR†: multivariate analysis including age, occupation, urbanization level, comorbidity of hyperlipidemia, diabetes, hypertension, benign prostatic hyperplasia, urinary calculi, obesity, asthma, coronary artery disease, chronic obstructive pulmonary disease, stroke, alcohol-related illness, prostatitis, and sexually transmitted diseases, and prostate cancer screening and diagnostic procedures with prostate biopsy and transurethral resection of the prostate.

## Discussion

To the best of our knowledge, no large-scale cohort studies have focused on the correlation between LUTI (cystitis and urethritis) and PCa. This study identified an association between PCa and a prior diagnosis of cystitis or urethritis. It may be argued that cystitis, urethritis and PCa represent overlapping symptoms, and that the increased risks are likely to be due to diagnostic confusion or misclassification. Therefore, we applied a sensitivity analysis. These results suggest that the LUTI cohort was associated with a significantly higher risk of developing PCa compared with the comparison cohort, despite the study population having at least a 3-year follow-up duration (Table 4). The frequency of medical visits for LUTI, which refers to severity of LUTI, is strongly correlated with the risk of subsequent PCa. These results are compatible with the finding that patients with cystitis or urethritis in Taiwan have an increased risk of developing PCa.

The mechanism underlying the association between LUTI and PCa is the focus of ongoing research. A possible explanation is that cystitis or urethritis cause bacterial prostatitis or chronic and asymptomatic inflammation of the prostate, resulting in prostatic carcinogenesis. It has been postulated that STDs also cause PCa by the same mechanism [22]. In particular, asymptomatic prostatic inflammation is common in adult males and this finding is supported by the fact that inflammatory cells were found in the prostate biopsy, or leukocytes found in semen analysis from patients without a history of prostatitis [23]. Biological studies have shown that inflamed tissue produces active oxygen and nitrogen radicals that increase the risk of cancer, by suppressing antitumor activity and stimulating carcinogenesis [24, 25]. Focal atrophy in the prostate is an inflammatory lesion, characteristic of infectious processes, with a reduced activity of glutathione S-transferase P1, an enzyme that protects the genome from oxidative damage. This lesion is thought to be a precursor of PCa [26]. In our analysis, cystitis or urethritis increased the risk of PCa, after adjusting for the effect of symptomatic prostatitis. This means that cystitis or urethritis may lead to asymptomatic prostatic inflammation and subsequently, carcinogenesis. The underlying mechanism of PCa and the role of asymptomatic prostatic inflammation require further investigation.

Epidemiological reports on the relationship between LUTI (cystitis or urethritis) and PCa risk are rare. Lightfoot et al. found that urinary tract infection was not associated with PCa in a population-based case-control study with 760 cases and 1632 controls [27]. However, in their analysis, urinary tract infection was not classified by infection site. Pelucchi et al. examined the association between cystitis and PCa risk in a hospital-based case-control study comprising 280 cases and 689 controls, and reported an odds ratios of 1.76 (95% CI = 1.07–2.91) [11]. However, the increased odds ratios were mainly restricted to participants who had suffered from an episode of cystitis within one year prior to the diagnosis of PCa (OR = 7.58, 95% CI = 1.42–40.45). Furthermore, there was only a trend for a higher risk of developing PCa in participants who experienced ≥3 episodes of cystitis than in those with no history of cystitis (OR = 1.83, 95% CI = 0.90–3.72). The authors concluded that the association between cystitis and PCa risk probably represented an early symptom of prostatic enlargement and suggested that cystitis had no role in PCa. The association between urethritis and PCa was investigated by Boehm and colleagues in a population-based case–control study which found that urethritis did not increase the risk of PCa (OR = 1.05, 95% CI = 0.84–1.30) [12].

Unlike previous case-control studies that did not include comorbidity data, we enrolled 14,273 patients with LUTI using a cohort study design, adjusting for possible confounding factors. The results indicate that the proportion of PCa patients, with or without cystitis, differs significantly over time, and the same phenomenon was observed in patients with or without urethritis (Fig 1). Furthermore, these results show a more than 9-fold increased risk of PCa among men with >5 medical visits per year for LUTI, compared to men with no history of LUTI. There is no significant difference in the PCa risk between participants with no history of LUTI and those with LUTI with an average of ≤3 medical visits per year. This may indicate the role of LUTI prevention or treatment in lowering the risk of PCa. The present data have been adjusted for comorbidities, such as BPH and prostatitis, to eliminate the possible effect due to prostate enlargement.

The large samples size obtained from our nationwide dataset strengthens the statistical power of our examination of the association between LUTI and PCa. Furthermore, our study was substantiated by the population-based design which allows extrapolation of results to other populations. Participants present a wide range of demographic characteristics. Therefore, we were able to perform stratified analyses based on age, occupation, and urbanization level. The ICD-9-CM code is considered reliable because it was recorded by clinical physician based on their diagnosis after reviewing the patient’s history, physical examination, lab data and possible image reports. For example, digital rectal examination, fractional urine examinations (urethral and bladder urine) and cytology of expressed prostatic secretions can help differentiate prostatitis from urethritis and cystitis. In clinical practice in Taiwan, we may check aforementioned examinations for the patients if the diagnosis of prostatitis is considered.

However, there are limitations to our findings. Firstly, the LHID does not contain detailed information on potential confounding factors, such as diet (intake of high saturated fat), smoking, alcohol consumption, high testosterone levels or family history of PCa. We only included comorbidities relevant to the LHID in Taiwan. We used smoking-related comorbidities (asthma, COPD, CAD, stroke, and hypertension), alcohol-related illness and diet-related comorbidities (obesity and hyperlipidemia) as indicators for smoking, alcohol consumption and dietary habits, respectively. Secondly, because there is no link between the LHID and the cancer registry, we did not have access to information regarding the stage or grade of PCa. Furthermore, the LHID provided no information on LUTI pathogens. This precluded us from performing any analysis involving these variables. Further study to evaluate the association between germs of LUTI and PCa is warranted. Thirdly, a screening bias may exist because of the possibility that a diagnosis of LUTI might increase the chance of further medical reviews or blood tests, which might increase incidental detection of PCa. In order to control for a potential screening bias, we analyzed screening and diagnostic procedures for PCa, including PCa biopsy and TURP.

In conclusion, our study supports the hypothesis that LUTI (cystitis or urethritis) is associated with an increased risk of PCa. In particular, subject with repeated medical visits for LUTI had a higher risk of developing PCa. Physicians should be aware of this association when assessing patients with LUTI. Further studies on the association between LUTI and PCa in other countries are warranted, especially where the prevalence of PCa is high.

## Supporting Information

S1 Checklist STROBE Checklist. (DOC)

## Abbreviations

BPH: benign prostatic hyperplasia
COPD: chronic obstructive pulmonary disease
CIs: confidence intervals
HRs: hazard ratios
ICD-9-CM: International Classification of Diseases, Ninth Revision, Clinical Modification
LUTI: lower urinary tract infection
LHID 2000: Longitudinal Health Insurance Database 2000
NHIRD: National Health Insurance Research Database
PCa: prostate cancer
STDs: sexually transmitted diseases
